# The TIDieR checklist will benefit the physical therapy profession

**DOI:** 10.1590/bjpt-rbf.2014.0182

**Published:** 2016

**Authors:** Tie Yamato, Chris Maher, Bruno Saragiotto, Anne Moseley, Tammy Hoffmann, Mark Elkins, Paula R. Camargo

**Affiliations:** 1 Musculoskeletal Division, The George Institute for Global Health, Sydney Medical School, The University of Sydney, Sydney, Australia; 2 Centre for Research in Evidence Based Practice, Faculty of Health Sciences and Medicine, Bond University, Queensland, Australia; 3 International Society of Physiotherapy Journal Editors, Camperdown, NSW, Australia; 4 Departament of Physical Therapy, Universidade Federal de São Carlos (UFSCar), São Carlos, SP, Brazil

Evidence-based practice involves physiotherapists incorporating high-quality clinical research on treatment efficacy into their clinical decision-making^1^. However, if clinical interventions are not adequately reported in the literature, physiotherapists face an important barrier to using effective interventions for their patients. Previous studies have reported that incomplete description of interventions is a problem in reports of randomised controlled trials in many health areas^2^ ^-^ ^4^. One of these studies^4^ examined 133 trials of non-pharmacological interventions. The experimental intervention was inadequately described in over 60% of the trials and descriptions of the control interventions were even worse.

A recent study^5^ evaluated the completeness of descriptions of the physical therapy interventions in a sample of 200 randomised controlled trials published in 2013. Overall, the interventions were poorly described. For the intervention groups, about one-quarter of the trials did not fulfil at least half of the criteria. Reporting for the control groups was even worse, with around three-quarters of trials not fulfilling at least half of the criteria. In other words, for the majority of the physical therapy trials, clinicians and researchers would be unable to replicate the interventions that were tested.

Describing a treatment may seem like a simple task but physical therapy interventions can be very complex. Some interventions are multi-modal, involving the use of manual techniques, consumable materials, equipment, education, training and feedback. Some interventions are tailored to each patient’s specific health state, including the patient’s immediate response to the application of the treatment. When the intervention involves a course of treatments, the intensity or dose may be progressed over time. The descriptions of physical therapy interventions in trial reports often do not capture all these components of the interventions or detail their complexity.

If researchers fail to comprehensively report all aspects of the interventions, the trial results cannot be incorporated into clinical practice or the intervention could be implemented incorrectly. Incorrect implementation may make the treatment ineffective, wasting the clinician’s and patient’s time and healthcare resources. Inadequate reporting of interventions also poses a barrier to incorporating a trial’s results into secondary research such as systematic reviews and clinical practice guidelines, as well as the usability of these resources. This means that the resources that were invested in undertaking the trial have been wasted. Such resources are extensive, including direct trial costs (e.g., payment of researchers, consumables), use of infrastructure (e.g., clinic space, equipment), human resources (e.g., ethics committee review, granting body review) and the goodwill of patients who agree to participate. Currently, there is a growing realisation that we need strategies to reduce waste in clinical research^6^. When the list of resources involved in a single study is considered, improving the reproducibility of interventions through better reporting could markedly reduce waste in research^7^.

The TIDieR checklist and guide were developed to improve the reporting of interventions in any evaluative study, including randomised trials^8^. The checklist contains 12 items and was developed as an extension to the CONSORT 2010^9^ and SPIRIT 2013^10^ statements to provide further guidance for authors on the key information to include in trial reports. TIDieR items include: name of the intervention; intervention rationale for essential elements; intervention materials and details about how to access them; description of the intervention procedures; details of intervention providers; mode of delivery of intervention; location of intervention delivery and key infrastructure; details about the number, duration, intensity and dose of intervention sessions; details of any intervention tailoring; any intervention modifications throughout the study; and details of intervention fidelity assessment, monitoring and level achieved. The TIDieR checklist will help improve the quality of intervention reporting more if it is used not only by study authors, but also journal editors, peer reviewers, ethics committees, and funding agencies. A copy of the checklist is available at: http://www.equator-network.org/reporting-guidelines/tidier/.

In summary, incomplete reporting of interventions in physical therapy studies is an important problem and we endorse the use of the TIDieR checklist as a potential solution. The responsibility for improving intervention reporting extends beyond the authors of individual trials to journal editors and others who can mandate the use of the TIDieR checklist to combat this problem. Mandating the use of the TIDieR checklist would guide authors to describe their interventions better and, consequently, help clinicians to use the interventions and researchers to synthesise and replicate the evidence.

At Brazilian Journal of Physical Therapy, the TIDieR statement will be incorporated into manuscript processing workflow from January 1st, 2017. Submitting authors are encouraged to use the TIDieR checklist to ensure that any interventions described in their manuscript are fully reported. However, submitting authors will not be required to submit the checklist. The editor will make an initial decision about the suitability of the manuscript for peer review. For manuscripts that are suitable for review, the editor will check the manuscript against the checklist to ensure that all items are fully reported. Manuscripts that do not report all relevant aspects of the intervention will be returned to the authors to address the gaps in reporting before the manuscript will progress to peer review.
